# 6′,7′-Dimeth­oxy-1′,2′-dihydro­spiro­[cyclo­hexane-1,2′-quinazolin]-4′(3′*H*)-one

**DOI:** 10.1107/S1600536811048732

**Published:** 2011-11-23

**Authors:** Li-Jun Zhang, Yang Song, Xiang-Ning Luo, Hui Li

**Affiliations:** a School of Chemistry and Chemical Engineering, Tianjin University of Technology, Tianjin 300384, People’s Republic of China

## Abstract

In the title compound, C_15_H_20_N_2_O_3_, prepared from the reaction of 2-amino-4,5-dimeth­oxy­benzonitrile and cyclo­hexa­none, the six-membered diaza ring assumes an envelope conformation. In the crystal, inversion dimers are formed by pairs of N—H⋯O hydrogen bonds. Futher N—H⋯O hydrogen bonds link the dimers into a two-dimensional structure parallel to (001).

## Related literature

For further information on the title compound, see: Chen *et al.* (2007 ▶). For related structures, see: Zhang *et al.* (2008 ▶). For the biological activity of related compounds, see: Hour *et al.* (2000 ▶).
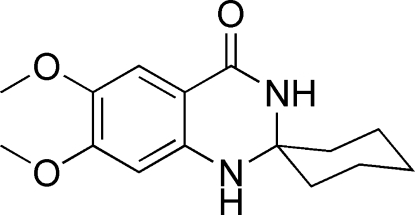


## Experimental

### 

#### Crystal data

C_15_H_20_N_2_O_3_
*M* *_r_* = 276.33
Monoclinic, 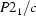

*a* = 11.667 (2) Å
*b* = 9.6376 (19) Å
*c* = 12.307 (3) Å
β = 101.02 (3)°
*V* = 1358.3 (5) Å^3^
*Z* = 4
Mo *K*α radiation
μ = 0.10 mm^−1^
*T* = 113 K
0.14 × 0.12 × 0.10 mm

#### Data collection

Rigaku Saturn diffractometer
Absorption correction: multi-scan (*CrystalClear*; Rigaku, 2004 ▶) *T*_min_ = 0.987, *T*_max_ = 0.991
16366 measured reflections
3224 independent reflections
2759 reflections with *I* > 2σ(*I*)
*R*_int_ = 0.031

#### Refinement

*R*[*F* ^2^ > 2σ(*F* ^2^)] = 0.036
*wR*(*F* ^2^) = 0.100
*S* = 1.09
3224 reflections
192 parameters
2 restraints
H atoms treated by a mixture of independent and constrained refinement
Δρ_max_ = 0.33 e Å^−3^
Δρ_min_ = −0.18 e Å^−3^

### 

Data collection: *CrystalClear* (Rigaku, 2004 ▶); cell refinement: *CrystalClear*; data reduction: *CrystalClear*; program(s) used to solve structure: *SHELXS97* (Sheldrick, 2008 ▶); program(s) used to refine structure: *SHELXL97* (Sheldrick, 2008 ▶); molecular graphics: *SHELXTL* (Sheldrick, 2008 ▶); software used to prepare material for publication: *SHELXL97*.

## Supplementary Material

Crystal structure: contains datablock(s) I, global. DOI: 10.1107/S1600536811048732/bg2428sup1.cif

Structure factors: contains datablock(s) I. DOI: 10.1107/S1600536811048732/bg2428Isup2.hkl

Supplementary material file. DOI: 10.1107/S1600536811048732/bg2428Isup4.cdx

Supplementary material file. DOI: 10.1107/S1600536811048732/bg2428Isup4.cml

Additional supplementary materials:  crystallographic information; 3D view; checkCIF report

## Figures and Tables

**Table 1:** Hydrogen-bond geometry (Å, °)

*D*—H⋯*A*	*D*—H	H⋯*A*	*D*⋯*A*	*D*—H⋯*A*
N2—H2⋯O3^i^	0.90 (1)	1.98 (1)	2.8801 (13)	176 (1)
N1—H1⋯O2^ii^	0.90 (1)	2.35 (1)	3.2267 (14)	168 (1)

Symmetry codes: (i) 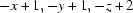; (ii) 
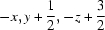
.

**Fig. 1.**
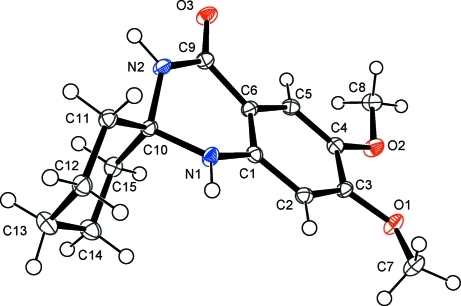
The molecular structure of (I), (Displacement ellipsoids drawn at a 50% probability level).

**Fig. 2.**
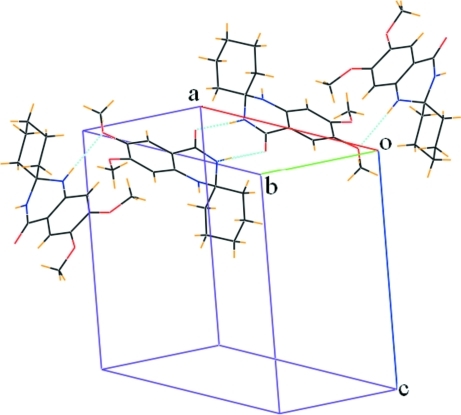
Packing diagram of (I), showing one dimer and the way they connect into (001) layers (H bonds in dashed lines).

## supplementary crystallographic information

### Comment

Dihydroquinazolin-4(3*H*)-ones possess a broad spectrum of biological and pharmaceutical activities, such as analgesic, antitumor, anticancer, diuretic, and herbicide activities (Hour *et al.*, 2000). 6,7-dimethoxyl-2,2-pentamethylene-1,2-dihydroquinazolin-4(3*H*)-one (I), a derivative of the most useful 1,2-dihydroquinazolinones (Chen *et al.*, 2007) was synthesized directly from the raction of 2-amino-4,5-dimethoxybenzonitrile and cyclohexanone. In order to further confirm its structure and illuminate the correlation of structural features with its biological activity, the single-crystal of title compound was determined by X-ray crystallographic analysis.

The molecular structure of (I) is shown in Fig.1. The six membered diaza ring assumes an envelope conformation. An H-bonded dimeric unit is formed around an inversion centre, through a N-H···O H-bond (Table 1, first entry); a second H-bond of the same type (Table 1, second entry) links dimers into a two-dimensional structure parallel to (001) (Fig 2).

### Experimental

The title compound was prepared from the reaction of 2-amino-4,5-methoxybenzonitrile (1 mmol) with cyclopentanone 1 mL in the catalysis of sodium ethoxide (0.2 mmol) at room temperature for 1 h. Then the product was precipitated spontaneously and separated by filtration. The pure desired compound I was recrystallized from ethanol in 91% yield.

Single crystals were obtained from a solution of ethanol by slow evaporation at room temperature.

### Refinement

Imino H atoms were located in a difference Fourier map and refined isotropically. Other H atoms were placed in calculated positiosn with C—H = 0.95 (aromatic ) or 0.98 Å (methylene), and refined in riding mode with U_iso_(H) = 1.2U_eq_(C) or 1.5U_eq_(C) (for methylene group).

### Crystal data

**Table:**

C_15_H_20_N_2_O_3_	*F*(000) = 592
*M**_r_* = 276.33	*D*_x_ = 1.351 Mg m^−^^3^
Monoclinic, *P*2_1_/*c*	Mo *K*α radiation, λ = 0.71073 Å
Hall symbol: -P 2ybc	Cell parameters from 3746 reflections
*a* = 11.667 (2) Å	θ = 1.8–27.9°
*b* = 9.6376 (19) Å	µ = 0.10 mm^−^^1^
*c* = 12.307 (3) Å	*T* = 113 K
β = 101.02 (3)°	Prism, colourless
*V* = 1358.3 (5) Å^3^	0.14 × 0.12 × 0.10 mm
*Z* = 4	

### Data collection

**Table:**

Rigaku Saturn diffractometer	3224 independent reflections
Radiation source: rotating anode	2759 reflections with *I* > 2σ(*I*)
confocal	*R*_int_ = 0.031
ω scans	θ_max_ = 27.9°, θ_min_ = 1.8°
Absorption correction: multi-scan (*CrystalClear*; Rigaku, 2004)	*h* = −15→14
*T*_min_ = 0.987, *T*_max_ = 0.991	*k* = −12→12
16366 measured reflections	*l* = −16→16

### Refinement

**Table:**

Refinement on *F*^2^	Secondary atom site location: difference Fourier map
Least-squares matrix: full	Hydrogen site location: inferred from neighbouring sites
*R*[*F*^2^ > 2σ(*F*^2^)] = 0.036	H atoms treated by a mixture of independent and constrained refinement
*wR*(*F*^2^) = 0.100	*w* = 1/[σ^2^(*F*_o_^2^) + (0.0565*P*)^2^ + 0.1742*P*] where *P* = (*F*_o_^2^ + 2*F*_c_^2^)/3
*S* = 1.09	(Δ/σ)_max_ < 0.001
3224 reflections	Δρ_max_ = 0.33 e Å^−^^3^
192 parameters	Δρ_min_ = −0.18 e Å^−^^3^
2 restraints	Extinction correction: *SHELXL97* (Sheldrick, 2008), Fc^*^=kFc[1+0.001xFc^2^λ^3^/sin(2θ)]^-1/4^
Primary atom site location: structure-invariant direct methods	Extinction coefficient: 0.073 (6)

### Special details

**Table:**

Experimental. Mp. 251–252°C. IR (KBr, cm^-1^): 3290, 3220, 2930, 2838, 1651, 1619, 1507; 1H NMR (400 MHz, DMSO-d_6_) δ_H_: 1.23–1.78 (10*H*, m, C5H10), 3.66(3*H*, s, OCH3), 3.74(3*H*, s, OCH3), 6.34 (1*H*, s, NH), 6.54 (1*H*, s, ArH), 7.07 (1*H*, s, ArH), 7.68 (1*H*, s, NH); 13 C NMR (100 MHz, DMSO-d_6_) δ_C_: 21.1 (2 C), 24.8, 37.0 (2 C), 55.4, 56.0, 68.2, 99.2, 106.0, 110.0, 141.1, 142.6, 153.9, 163.4; MS (ESI): m/*z* (%) = 277.2 (100) [*M*+H]+; Anal. Calcd. for C15H20N2O3: C 65.20, H 7.30, N 10.14; found C 64.98, H 7.80, N 9.52.
Geometry. All e.s.d.'s (except the e.s.d. in the dihedral angle between two l.s. planes) are estimated using the full covariance matrix. The cell e.s.d.'s are taken into account individually in the estimation of e.s.d.'s in distances, angles and torsion angles; correlations between e.s.d.'s in cell parameters are only used when they are defined by crystal symmetry. An approximate (isotropic) treatment of cell e.s.d.'s is used for estimating e.s.d.'s involving l.s. planes.
Refinement. Refinement of *F*^2^ against ALL reflections. The weighted *R*-factor *wR* and goodness of fit *S* are based on *F*^2^, conventional *R*-factors *R* are based on *F*, with *F* set to zero for negative *F*^2^. The threshold expression of *F*^2^ > σ(*F*^2^) is used only for calculating *R*-factors(gt) *etc*. and is not relevant to the choice of reflections for refinement. *R*-factors based on *F*^2^ are statistically about twice as large as those based on *F*, and *R*- factors based on ALL data will be even larger.

### Fractional atomic coordinates and isotropic or equivalent isotropic displacement parameters (Å^2^)

**Table:**

	*x*	*y*	*z*	*U*_iso_*/*U*_eq_	
O1	−0.16045 (6)	0.43728 (8)	0.80668 (6)	0.0203 (2)	
O2	−0.06468 (6)	0.26156 (8)	0.95294 (6)	0.0205 (2)	
O3	0.37247 (6)	0.40011 (8)	1.03202 (6)	0.0200 (2)	
N1	0.21715 (7)	0.61646 (9)	0.76365 (7)	0.0153 (2)	
N2	0.38438 (7)	0.54041 (10)	0.88635 (7)	0.0158 (2)	
C1	0.14797 (9)	0.52723 (11)	0.81032 (8)	0.0134 (2)	
C2	0.02567 (9)	0.52808 (11)	0.77773 (8)	0.0145 (2)	
H2A	−0.0099	0.5871	0.7215	0.017*	
C3	−0.04157 (9)	0.44150 (11)	0.82915 (8)	0.0151 (2)	
C4	0.01161 (9)	0.34735 (11)	0.91200 (8)	0.0152 (2)	
C5	0.13085 (9)	0.34881 (11)	0.94579 (8)	0.0153 (2)	
H5	0.1660	0.2894	1.0020	0.018*	
C6	0.20027 (9)	0.43927 (11)	0.89624 (8)	0.0142 (2)	
C7	−0.22059 (9)	0.54426 (13)	0.73899 (10)	0.0226 (3)	
H7A	−0.1917	0.6330	0.7673	0.034*	
H7B	−0.3026	0.5382	0.7394	0.034*	
H7C	−0.2080	0.5337	0.6646	0.034*	
C8	−0.01742 (10)	0.18413 (12)	1.04951 (9)	0.0199 (2)	
H8A	0.0375	0.1175	1.0321	0.030*	
H8B	−0.0793	0.1368	1.0756	0.030*	
H8C	0.0214	0.2459	1.1059	0.030*	
C9	0.32557 (9)	0.45515 (11)	0.94320 (8)	0.0152 (2)	
C10	0.33946 (9)	0.57909 (11)	0.77095 (8)	0.0134 (2)	
C11	0.40780 (9)	0.70426 (11)	0.74216 (8)	0.0152 (2)	
H11A	0.4907	0.6843	0.7621	0.018*	
H11B	0.3916	0.7831	0.7859	0.018*	
C12	0.37805 (9)	0.74296 (11)	0.61959 (9)	0.0170 (2)	
H12A	0.2979	0.7751	0.6015	0.020*	
H12B	0.4283	0.8182	0.6051	0.020*	
C13	0.39356 (10)	0.61926 (13)	0.54656 (9)	0.0222 (3)	
H13A	0.4756	0.5946	0.5580	0.027*	
H13B	0.3685	0.6448	0.4694	0.027*	
C14	0.32319 (10)	0.49419 (12)	0.57250 (9)	0.0207 (3)	
H14A	0.2405	0.5153	0.5532	0.025*	
H14B	0.3387	0.4156	0.5282	0.025*	
C15	0.35496 (9)	0.45662 (11)	0.69493 (9)	0.0164 (2)	
H15A	0.4356	0.4258	0.7118	0.020*	
H15B	0.3062	0.3801	0.7098	0.020*	
H1	0.1830 (11)	0.6677 (13)	0.7058 (9)	0.029 (4)*	
H2	0.4604 (8)	0.5564 (14)	0.9151 (11)	0.032 (4)*	

### Atomic displacement parameters (Å^2^)

**Table:**

	*U*^11^	*U*^22^	*U*^33^	*U*^12^	*U*^13^	*U*^23^
O1	0.0111 (4)	0.0221 (4)	0.0265 (4)	−0.0010 (3)	0.0004 (3)	0.0047 (3)
O2	0.0161 (4)	0.0231 (4)	0.0215 (4)	−0.0058 (3)	0.0013 (3)	0.0065 (3)
O3	0.0163 (4)	0.0240 (4)	0.0179 (4)	−0.0021 (3)	−0.0015 (3)	0.0081 (3)
N1	0.0115 (4)	0.0175 (5)	0.0169 (4)	0.0020 (3)	0.0028 (3)	0.0055 (4)
N2	0.0115 (4)	0.0201 (5)	0.0145 (4)	−0.0026 (4)	−0.0007 (3)	0.0032 (4)
C1	0.0146 (5)	0.0130 (5)	0.0130 (5)	−0.0005 (4)	0.0036 (4)	−0.0014 (4)
C2	0.0158 (5)	0.0141 (5)	0.0129 (5)	0.0015 (4)	0.0015 (4)	0.0000 (4)
C3	0.0116 (5)	0.0165 (5)	0.0163 (5)	−0.0009 (4)	0.0007 (4)	−0.0036 (4)
C4	0.0162 (5)	0.0141 (5)	0.0158 (5)	−0.0035 (4)	0.0041 (4)	−0.0013 (4)
C5	0.0171 (5)	0.0147 (5)	0.0136 (5)	−0.0001 (4)	0.0016 (4)	0.0011 (4)
C6	0.0131 (5)	0.0147 (5)	0.0141 (5)	−0.0003 (4)	0.0013 (4)	−0.0005 (4)
C7	0.0140 (5)	0.0258 (6)	0.0260 (6)	0.0026 (4)	−0.0012 (4)	0.0029 (5)
C8	0.0219 (6)	0.0187 (5)	0.0185 (5)	−0.0053 (4)	0.0029 (4)	0.0029 (4)
C9	0.0149 (5)	0.0148 (5)	0.0157 (5)	−0.0007 (4)	0.0019 (4)	0.0003 (4)
C10	0.0108 (5)	0.0156 (5)	0.0134 (5)	0.0002 (4)	0.0011 (4)	0.0024 (4)
C11	0.0139 (5)	0.0152 (5)	0.0167 (5)	−0.0022 (4)	0.0030 (4)	0.0003 (4)
C12	0.0173 (5)	0.0172 (5)	0.0172 (5)	−0.0012 (4)	0.0047 (4)	0.0029 (4)
C13	0.0266 (6)	0.0246 (6)	0.0173 (5)	−0.0009 (5)	0.0089 (5)	0.0000 (4)
C14	0.0243 (6)	0.0207 (6)	0.0173 (5)	−0.0013 (5)	0.0049 (4)	−0.0036 (4)
C15	0.0162 (5)	0.0138 (5)	0.0195 (5)	0.0009 (4)	0.0038 (4)	0.0002 (4)

### Geometric parameters (Å, °)

**Table:**

O1—C3	1.3619 (12)	C7—H7B	0.9600
O1—C7	1.4233 (13)	C7—H7C	0.9600
O2—C4	1.3789 (12)	C8—H8A	0.9600
O2—C8	1.4223 (13)	C8—H8B	0.9600
O3—C9	1.2438 (13)	C8—H8C	0.9600
N1—C1	1.3777 (13)	C10—C11	1.5246 (14)
N1—C10	1.4577 (13)	C10—C15	1.5381 (14)
N1—H1	0.895 (8)	C11—C12	1.5280 (14)
N2—C9	1.3487 (14)	C11—H11A	0.9700
N2—C10	1.4650 (13)	C11—H11B	0.9700
N2—H2	0.903 (9)	C12—C13	1.5246 (16)
C1—C6	1.4009 (14)	C12—H12A	0.9700
C1—C2	1.4064 (14)	C12—H12B	0.9700
C2—C3	1.3791 (15)	C13—C14	1.5261 (16)
C2—H2A	0.9300	C13—H13A	0.9700
C3—C4	1.4164 (15)	C13—H13B	0.9700
C4—C5	1.3737 (15)	C14—C15	1.5250 (15)
C5—C6	1.4060 (14)	C14—H14A	0.9700
C5—H5	0.9300	C14—H14B	0.9700
C6—C9	1.4735 (14)	C15—H15A	0.9700
C7—H7A	0.9600	C15—H15B	0.9700
			
C3—O1—C7	117.79 (9)	O3—C9—C6	122.49 (10)
C4—O2—C8	116.47 (8)	N2—C9—C6	115.13 (9)
C1—N1—C10	117.69 (8)	N1—C10—N2	106.68 (8)
C1—N1—H1	118.0 (9)	N1—C10—C11	109.84 (8)
C10—N1—H1	117.8 (9)	N2—C10—C11	108.70 (8)
C9—N2—C10	122.33 (9)	N1—C10—C15	112.31 (9)
C9—N2—H2	117.4 (9)	N2—C10—C15	109.43 (8)
C10—N2—H2	118.6 (9)	C11—C10—C15	109.77 (8)
N1—C1—C6	119.15 (9)	C10—C11—C12	113.10 (8)
N1—C1—C2	121.38 (9)	C10—C11—H11A	109.0
C6—C1—C2	119.37 (9)	C12—C11—H11A	109.0
C3—C2—C1	120.05 (10)	C10—C11—H11B	109.0
C3—C2—H2A	120.0	C12—C11—H11B	109.0
C1—C2—H2A	120.0	H11A—C11—H11B	107.8
O1—C3—C2	124.84 (10)	C13—C12—C11	111.16 (9)
O1—C3—C4	114.59 (9)	C13—C12—H12A	109.4
C2—C3—C4	120.57 (9)	C11—C12—H12A	109.4
C5—C4—O2	125.64 (9)	C13—C12—H12B	109.4
C5—C4—C3	119.36 (10)	C11—C12—H12B	109.4
O2—C4—C3	115.00 (9)	H12A—C12—H12B	108.0
C4—C5—C6	120.60 (10)	C12—C13—C14	111.41 (9)
C4—C5—H5	119.7	C12—C13—H13A	109.3
C6—C5—H5	119.7	C14—C13—H13A	109.3
C1—C6—C5	119.92 (9)	C12—C13—H13B	109.3
C1—C6—C9	119.31 (9)	C14—C13—H13B	109.3
C5—C6—C9	120.32 (9)	H13A—C13—H13B	108.0
O1—C7—H7A	109.5	C15—C14—C13	110.82 (9)
O1—C7—H7B	109.5	C15—C14—H14A	109.5
H7A—C7—H7B	109.5	C13—C14—H14A	109.5
O1—C7—H7C	109.5	C15—C14—H14B	109.5
H7A—C7—H7C	109.5	C13—C14—H14B	109.5
H7B—C7—H7C	109.5	H14A—C14—H14B	108.1
O2—C8—H8A	109.5	C14—C15—C10	112.59 (9)
O2—C8—H8B	109.5	C14—C15—H15A	109.1
H8A—C8—H8B	109.5	C10—C15—H15A	109.1
O2—C8—H8C	109.5	C14—C15—H15B	109.1
H8A—C8—H8C	109.5	C10—C15—H15B	109.1
H8B—C8—H8C	109.5	H15A—C15—H15B	107.8
O3—C9—N2	122.27 (9)		
			
C10—N1—C1—C6	24.75 (14)	C10—N2—C9—O3	165.20 (10)
C10—N1—C1—C2	−158.81 (9)	C10—N2—C9—C6	−18.58 (14)
N1—C1—C2—C3	−177.33 (9)	C1—C6—C9—O3	165.95 (10)
C6—C1—C2—C3	−0.90 (15)	C5—C6—C9—O3	−6.30 (16)
C7—O1—C3—C2	−10.82 (15)	C1—C6—C9—N2	−10.26 (14)
C7—O1—C3—C4	169.32 (9)	C5—C6—C9—N2	177.49 (9)
C1—C2—C3—O1	177.71 (9)	C1—N1—C10—N2	−48.06 (12)
C1—C2—C3—C4	−2.43 (15)	C1—N1—C10—C11	−165.70 (9)
C8—O2—C4—C5	10.36 (15)	C1—N1—C10—C15	71.83 (11)
C8—O2—C4—C3	−168.79 (9)	C9—N2—C10—N1	46.21 (13)
O1—C3—C4—C5	−176.12 (9)	C9—N2—C10—C11	164.60 (9)
C2—C3—C4—C5	4.01 (15)	C9—N2—C10—C15	−75.52 (12)
O1—C3—C4—O2	3.09 (13)	N1—C10—C11—C12	−70.47 (11)
C2—C3—C4—O2	−176.78 (9)	N2—C10—C11—C12	173.15 (8)
O2—C4—C5—C6	178.66 (9)	C15—C10—C11—C12	53.49 (11)
C3—C4—C5—C6	−2.21 (15)	C10—C11—C12—C13	−54.58 (12)
N1—C1—C6—C5	179.18 (9)	C11—C12—C13—C14	54.73 (12)
C2—C1—C6—C5	2.67 (15)	C12—C13—C14—C15	−55.51 (12)
N1—C1—C6—C9	6.90 (14)	C13—C14—C15—C10	55.82 (12)
C2—C1—C6—C9	−169.61 (9)	N1—C10—C15—C14	68.32 (11)
C4—C5—C6—C1	−1.10 (15)	N2—C10—C15—C14	−173.39 (8)
C4—C5—C6—C9	171.11 (9)	C11—C10—C15—C14	−54.19 (11)

### Hydrogen-bond geometry (Å, °)

**Table:**

*D*—H···*A*	*D*—H	H···*A*	*D*···*A*	*D*—H···*A*
N2—H2···O3^i^	0.90 (1)	1.98 (1)	2.8801 (13)	176.(1)
N1—H1···O2^ii^	0.90 (1)	2.35 (1)	3.2267 (14)	168.(1)

Symmetry codes: (i) −*x*+1, −*y*+1, −*z*+2; (ii) −*x*, *y*+1/2, −*z*+3/2.
